# Once Daily High Dose Tigecycline Is Optimal: Tigecycline PK/PD Parameters Predict Clinical Effectiveness

**DOI:** 10.3390/jcm7030049

**Published:** 2018-03-09

**Authors:** Jeffrey Baron, Shuntao Cai, Natalie Klein, Burke A. Cunha

**Affiliations:** 1 Departments of Pharmacy, Roswell Park Comprehensive Cancer Center, Buffalo, NY 14263, USA; jeffreyibaron@gmail.com; 2 Departments of Oncology, Winthrop-University Hospital, Mineola, NY 11501, USA; scai@winthrop.org; 3 Infectious Disease Division, Winthrop-University Hospital, Mineola, NY 11501, USA; nklein@nyuwinthrop.org; 4 School of Medicine, State University of New York, Stony Brook, NY 11794-8434, USA

**Keywords:** tigecycline pharmacokinetics, tigecycline effectiveness, high dose once daily tigecycline, dose dependent susceptibility, tigecycline susceptible MDR pathogens

## Abstract

Objective: The clinical effectiveness of tigecycline depends on appropriate use, and PK/PD (pharmacokinetic/pharmacodynamic) parameters related to dose and dosing interval. Methods: In our 600-bed university-affiliated teaching hospital, we conducted a tigecycline efficacy review over a three-month period in 34 evaluable patients. Parameters assessed included clinical response, cure or treatment failure, once daily as q12h dosing, maintenance dosing, high dose vs. standard loading regimens, adverse effects, and the effect of infectious disease consultation on outcomes. Results: We found once daily high dose tigecycline (HDT) was highly effective in treating serious systemic infections due to MDR Gram-positive/negative pathogens as well as *C. difficile* colitis. Adverse effects were infrequent and limited to mild nausea/vomiting. Once daily HDT was highly effective, and the few treatment failures were related to suboptimal/split dosing regimens. Conclusion: Once daily HDT was highly effective when used to treat susceptible pathogens and when optimally dosed, i.e., 200–400 mg (IV) loading dose ×1, followed by a once daily maintenance dose of 100–200 mg (IV) q24h.

## 1. Introduction

The incidence of multidrug-resistant pathogens: methicillin-resistant *Staphylococcus aureus* (MRSA), *S. faecium*/vancomycin-resistant *enterococci* (VRE), *Pseudomonas aeruginosa*, *Acinetobacter baumannii* and extended spectrum β-lactamase (ESBL)-producing Enterobacteriaceae has risen in recent years. These multidrug-resistant pathogens present therapeutic challenges due to the limited number of antimicrobial agents with activity against multidrug-resistant pathogens. Furthermore, multidrug-resistant pathogens, if ineffectively treated, may result in poor patient outcomes. As a result, there is a need for antimicrobial agents that do not predispose to resistance and remain effective against multidrug-resistant pathogens; tigecycline is such an antimicrobial.

Tigecycline is a glycylcycline antibiotic with activity against nearly all Gram-positive Gram-negative and anaerobic pathogens except *Proteus* sp. and *P. aeruginosa*. Specifically, tigecycline has in vitro activity against *Enterococcus faecalis/*vancomycin sensitive *enterococci* (VSE), *Enterococcus faecium* (VRE), *Staphylococcus aureus* (MSSA/MRSA), *Streptococcus pyogenes*, *Streptococcus agalactiae*, *Streptococcus anginosus group*, *Streptococcus pneumoniae, Citrobacter freundii*, *Enterobacter cloacae*, *Escherichia coli*, *Haemophilus influenzae*, *Klebsiella oxytoca*, *Klebsiella pneumoniae*, *Peptostreptococcus* sp., *Bacteroides fragilis*, *Bacteroides thetaiotaomicron*, *Bacteroides uniformis*, *Bacteroides vulgatus*, *Clostridium perfringens and Legionella pneumophila*. Tigecycline is FDA-approved for treatment of complicated skin and skin structure infections (cSSSIs), complicated intra-abdominal infections (cIAIs) and community-acquired bacterial pneumonia (CAP), in those 18 years or older [1,2].

According to the package insert (PI) prescribing information and labeling, tigecycline recommended dosing is 100 mg (IV) loading dose ×1 followed by 50 mg (IV) q12h, i.e., standard dose tigecycline (SDT). Pharmacokinetic studies (PK) have shown tigecycline has a serum half-life (t ½) of 27 h following a single dose and 42 h following multiple doses [2,3,4]. Because of its long half-life (t ½), some centers, including ours, have implemented a high dose strategy for tigecycline, using doses outside the recommended label. At our center, high dose tigecycline (HDT) is defined using once daily strategy, with a 200 mg (IV) loading dose, followed by 100 mg (IV) q24h. When given as a single daily dose, like doxycycline, tigecycline exhibits concentration-dependent kinetics whereas using split-daily dosing, tigecycline exhibits time-dependent kinetics. Based on the pharmacokinetics and pharmacodynamics, along with a prolonged post-antibiotic effect, tigecycline dosing should be optimized to a high dose strategy to provide optimal antibacterial effects [5,6]. Cunha and colleagues first reported once HDT is effective and has become the preferred dosing regimen at Winthrop-University Hospital for treating serious systemic infections due to susceptible and multidrug-resistant (MDR) pathogens [7,8].

Tigecycline, a novel analog of minocycline, has been shown to be unaffected by mechanisms of tetracycline resistance explaining its usefulness as an antimicrobial MDR infections. Current experience and in vitro data support tigecycline use for MDR infections, including MRSA, VRE, multidrug-resistant *Klebsiella pneumoniae* and carbapenemase-resistant Enterobacteriaciae (CRE) [7,8,9,10]. Furthermore, tigecycline is protective against *Clostridium difficile* diarrhea and tigecycline has been used in severe *Clostridium difficile* colitis [3]. For serious systemic infections, tigecycline has been shown to be safe and effective. Clinically, while safe and effective, tigecycline carries a black box warning of treatment-related mortality [2]. This increased risk of all-cause mortality was reported in a meta-analysis which compared tigecycline use to comparator antibiotics and found a risk difference of 0.6% (95% CI 0.1–1.2). However, the cause of the risk difference was in part due to suboptimal (package insert) dosing [4]. Also, in this study tigecycline failures mostly in infections due to organisms beyond its natural spectrum, i.e., tigecycline has little/no activity against *Proteus* sp. *P. aeruginosa* [7,8,9]. Additional studies are needed to assess efficacy of once daily dosing tigecycline for use in serious systemic infections. Therefore, a retrospective efficacy evaluation was undertaken to assess the safety and efficacy of SDT vs HDT tigecycline at Winthrop University Hospital, a 600-bed university affiliated teaching hospital.

## 2. Methods

A retrospective efficacy study of recent tigecycline use over a three-month was undertaken at Winthrop-University Hospital. 34 patients that received tigecycline were included for review. Patients receiving ≤1 day of tigecycline were excluded due insufficient length of therapy. All patient information was de-identified and stored on secure computers for proper protection. Appropriateness of tigecycline was defined by composite endpoint of indications, treatment outcomes and the presence or absence of infectious disease consult for guidance in antimicrobial therapy. Treatment outcomes were defined as success, failure, treatment discontinuation due to adverse effects and/or death. Treatment failure was defined persistence of signs/symptoms of infection despite antimicrobial therapy and/or use of tigecycline when other antimicrobials were available. Treatment success was defined as resolution of signs/symptoms of infection, microbiological cure (negative cultures after tigecycline use), and/or use of tigecycline when other antimicrobials were not appropriate. Additional endpoints include incidence of adverse effects and doses used.

## 3. Results

A total of 34 cases were identified during the three-month study period with 50% of the cases being males and 50% being female and a mean age of 67 years. Mean hospital length of stay (LOS) was 23.3 days and mean length of therapy for tigecycline was 6.5 days. Our study found that tigecycline was being used for various indications: cSSSIs (41.2%), *C. difficile* (20.6%), MDR *Klebsiella pneumoniae* (11.8%), CAP/HAP (8.8%), cIAIs (5.9%), VRE (2.9%), post-operatively (2.9%) and for unknown reasons (2.9%). For the primary endpoint of treatment outcomes (Table 1) there testament successes (79.4%), three failures (8.8%), two treatment discontinuations (5.9%) and there were two deaths (5.9%). The deaths were due to septic shock with fulminant infections; it was felt that deaths were due to complications of septic shock and not directly from infection. The secondary endpoints were also evaluated in our study (Table 2). Regarding loading doses (LD), a loading dose was not used 73.5% of the time. When a loading dose was used, the most common LD used was either 400 mg (IV) × 1 (11.8%) or 200 mg (IV) × 1. The most common maintenance dose (MD) was 100 mg (IV) q24h (64.7%), or 50 mg q12h (23.5%). In 28/34 cases (82.4%), infectious disease consultants helped optimize tigecycline therapy. There were 6 cases (17.6%) of nausea/vomiting in our study group. For the composite endpoint of appropriateness of tigecycline therapy, tigecycline use was appropriately used in 28/34 cases (82.4%).

## 4. Discussion

Based on the results of this efficacy study, tigecycline use at Winthrop University Hospital was appropriate as confirmed by high rates of treatment success (79.4%). These high rates of treatment success were due to once daily HDT and infectious disease consultation in the majority of cases (82.4%) to optimize tigecycline therapy. Package insert (PI) prescribing information which recommends tigecycline should only be used when alternative options are not appropriate [2,11,12,13,14,15,16,17,18]. It is important to note (that despite high rates of treatment success), there were 6 cases of inappropriate tigecycline use. Due to broad spectrum of coverage and the ability to give tigecycline in renal insufficiency, tigecycline is a useful antimicrobial for CSSSI diabetic foot infections, but the first case involved a diabetic foot infection for which tigecycline is not approved. The second case of inappropriate use involved a cSSSI in which the patient was on ceftaroline and was then switched to tigecycline; the patient had improved, and it was unclear why the patient was switched to tigecycline. Because of the unnecessary switch to tigecycline, when tigecycline therapy was not needed, was considered inappropriate use. The third inappropriate case was tigecycline use for “*C. difficile* colitis”, in which *C. difficile* toxin was negative and CT scan showed no evidence of colitis. There was a fourth inappropriate case in which the patient had a urinary tract infection (UTI) sensitive to ceftriaxone, meropenem, ceftazidime, cefepime, levofloxacin and trimethoprim-sulfamethoxazole. Tigecycline use was considered inappropriate since alternative options were available and appropriate for use. The fifth case involving inappropriate use of tigecycline was the post-operative antimicrobial prophylacis use; this patient was admitted for a poor functioning peritoneal dialysis catheter secondary to hernia. The patient underwent surgical repair and the tigecycline was ordered by nephrology, with no infectious disease consult or rationale for tigecycline use.

In reviewing the indications for tigecycline use, the most common indications were cSSSIs (approved indication), MDR *Klebsiella pneumoniae* infections (off-label use) and *C. difficile* infections (off-label use). There is a wealth of literature that supports the efficacy of tigecycline for MDR infections, e.g., MRSA, VRSA, *Klebsiella pneumoniae* and *Acinetobacter* [1,7,8,9,18]. In addition, there is a considerable literature to suggest that tigecycline may be used as monotherapy or in combination therapy for severe *C. difficile* colitis [10,11,12,13,19]. For cases of *C. difficile* colitis or MDR Gram negative infections, use of once daily HDT was highly effective in all but one case. Our study results support using once daily HDT for MDR infections, serious systemic infections and infections due to severe *C. difficile* colitis.

As mentioned, antibiotic resistance to some antimicrobial agents is increasing. Bacterial resistance can be classified into either natural/intrinsic resistance, acquired relative/resistance or absolute/resistance high levels. When an antibiotic is classified as naturally/intrinsically resistant means it has no inherent activity against certain pathogens. Acquired resistance refers to a previously susceptible pathogen that is no longer susceptive to an antibiotic, e.g., ampicillin-resistant *H. influenzae*. Non-susceptible or “relative resistance” may be overcome using high dose regimens. Absolute resistance is the term for resistance that cannot be overcome with higher than usual doses, e.g., gentamicin-resistant *P. aeruginosa*. Resistance is also partly related to volume or duration of use. Antibiotics can be considered as “high resistance potential” or “low resistance potential” antibiotics. “high resistance potential” antibiotics includes antibiotics which lead to increased multidrug-resistant pathogens, e.g., ceftazidime, imipenem, gentamicin, ciprofloxacin. When dosed properly, “low resistance potential” antimicrobials retain a “low resistance potential” and intensive/prolonged use do not predispose to resistance.

Based on reported worldwide experience to date, tigecycline has a “low resistance potential”. However, inadequately dosed tigecycline may be associated with treatment failure and/or resistance [8,19]. Recent evidence has shown that SDT for serious Gram-negative infections, using PI maintenance dosing (MD), i.e., 50 mg (IV) q12h has been associated with suboptimal results [20,21]. A randomized controlled phase 2 trial by Ramirez et al. and a retrospective study by De Pascale et al. have shown for serious MDR Gram negative infections, the use of HDT, i.e., 100 mg (IV) q12h was associated with better outcomes compared to SDT (PI based dosing) of 50 mg (IV) q12h [20,21,22]. A systematic review by Falagas et al. highlighted the need for once daily HDT for serious MDR Gram negative infections [22]. In our efficacy study, the use of SDT (PI recommended dosing) was associated with treatment failure. The results of our efficacy study are in agreement with our previous and ongoing experience and the recent literature that based on PK/PD considerations and clinical experience that HDT provides optimal effectiveness since tigecycline displays concentration dependent killing. Because relative resistance is, in part, concentration dependent, once daily HDT is optimal for treatment of serious MDR Gram negative infections and overcome “relative resistance” [7,8,9,17]. Optimal PK based once daily HDT predicts treatment success [18,19,20,21,22]. Our experience is in agreement with others, i.e., suboptimal dosing predicts treatment failures [15,18].

Regarding adverse effects, tigecycline was only associated with mild nausea and vomiting. The incidence of nausea/vomiting in our study was 17.6%. Prescribing PI information states the incidence of nausea as 26% (severe 1%) and vomiting as 18% (severe 1%) [1]. For management of nausea/vomiting associated with tigecycline, we double the infusion time and dilute the drug in twice the usual volume [9]. The incidence of nausea/vomiting in our study was well below that listed in PI prescribing information. No other adverse events occurred. In this study tigecycline, was safe, and nausea/vomiting was mild and easily eliminated as described.

This small retrospective single center study had several limitations, including small number of cases and the microbiology laboratory at Winthrop-University Hospital does not routinely test for tigecycline susceptibilities, and for this reason, susceptibility data was not included. While twice daily SDT dosing would represent a control, because there were so few cases of SDT dosing, it is not possible to have a control for this study. As a result of not having a control, there was no post-hoc analysis to compare once daily HDT dosing to the twice SDT daily dosing. Lastly, microbiological data post initiation of tigecycline was not available for every case; therefore, it was not included in order to be consistent across the entire study.

## 5. Conclusions

In conclusion, this small study showed once daily HDT was safe and highly effective. Additionally, these results as well as others and our ongoing experience adds to the published literature that tigecycline is safe and effective for the treatment of severe *C. difficile* infections. We have also shown that tigecycline’s optimal effectiveness was highest when given in high dose once daily. Without infectious disease consultation, tigecycline was either used inappropriately or suboptimally dosed.

While more published experience is needed to further determine the effectiveness of high dose once daily tigecycline dosing in treating various serious systemic infections, our recent and ongoing experience at Winthrop-University Hospital has demonstrated that a PK/PD based tigecycline once daily regimen is optimal and PI based low/split dosing was suboptimal and was associated with treatment failures [23,24,25,26]. Once daily HDT is safe and optimally effective. Optimal, once daily high dose tigecycline dosing is based on PK/PD considerations, and the clinical experience of others and ourselves. Furthermore, PI based dosing should be replaced by PK/PD based dosing, i.e., once daily, not q12h, tigecycline. We conclude that once daily HDT may be preferred dosing regimen, based on current literature, to achieve maximal effectiveness for serious systemic infections, and MDR Gram negative infections including UTIs.

## Figures and Tables

**Table 1:** Winthrop University Hospital’s recent tigecycline experience: primary outcomes.

**Indication**	***n* = cases (%)**
cSSSIs	14 (41.2%)
*C. difficile* Infections	7 (20.6%)
MDR Klebsiella Infections	4 (11.8%)
Pneumonias	3 (8.8%)
cIAIs	2 (5.9%)
VRE Infections	2 (5.9%)
Post-operative infections	1 (2.9%)
Unknown	1 (2.9%)
**Treatment Outcomes by Indication**	
cSSSIs—Success	13 (92.9%)
cSSSIs—Failures	1 (7.1%)
*C. difficile* Infections—Success	7 (85.7%)
*C. difficile* Infection—Failure	0 (0%)
MDR Klebsiella Infections—Success	3 (75%)
MDR Klebsiella Infections—Failure	1 (25%)
Pneumonias—Success	3 (100%)
Pneumonias—Failure	0 (0%)
cIAIs—Success	2 (100%)
cIAIs—Failure	0 (0%)
Post-operative infections—Success	1 (100%)
Post-operative infections—Failure	0 (0%)
**Treatment Outcomes HDT**	
Success	23 (95.8%)
Failure	1 (4.2%)
**Treatment Outcomes SDT**	
Success	4 (66.7%)
Failure	2 (33.3%)
**Overall Treatment Outcomes**	
Success	27 (79.4%)
Failure †	3 (8.8%)
Treatment Discontinuation due to ADE	2 (5.9%)
Death *	2 (5.9%)
**Appropriate Use**	
Yes	28 (82.4%)
No	6 (17.6%)

cSSSIs = complicated skin & skin structure infections; MDR = multi-drug-resistant; cIAIs = complicated intra-abdominal infections; ADE = adverse drug effects; VRE = vancomycin-resistant *enterococci*; * = unrelated to infection or tigecycline; † Treatment failure associated with using standard dosing tigecycline (SDT).

**Table 2:** Winthrop University Hospital’s recent tigecycline experience: secondary endpoints.

**Loading Doses Used**	***n* = cases (%)**
None	25 (73.5%)
100 mg (IV) × 1	3 (8.8%)
200 mg (IV) × 1	2 (5.9%)
400 mg(IV) × 1	4 (11.8%)
**Maintenance Doses Used**	
50 mg (IV) q12h	8 (23.5%)
100 mg (IV) q24h	22 (64.7%)
200 mg (IV) q24h	4 (11.8%)
**Infectious Disease Consultation**	
Yes	28 (82.4%)
No	6 (17.6%)
**Incidence of Nausea/Vomiting**	
Yes	6 (17.6%)
No	28 (82.4%)

## References

[1] Wyeth Pharmaceutics (2005). Tygacil (Tigecycline) for Injection [Package Insert].

[2] Schafer J.J., Goff D.A. (2008). Establishing the role of tigecycline in an era of antimicrobial resistance. Exp. Rev. Anti-Infect. Ther..

[3] Rello J. (2005). Pharmacokinetics, pharmacodynamics, safety and tolerability of tigecycline. J. Chemother..

[4] Barbour A., Schmidt S., Ma B., Schiefelbein L., Rand K.H., Burkhardt O., Derendorf H. (2009). Clinical pharmacokinetics and pharmacodynamics of tigecycline. Clin. Pharm..

[5] Cunha B.A. (2015). Antibiotic Essentials.

[6] Cunha B.A., Domenico P., Cunha C.B. (2000). Pharmacodynamics of doxycycline. Clin. Microbiol. Infect..

[7] Cunha B.A. (2007). Once-daily tigecycline therapy of multidrug-resistant and non-multidrug-resistant gram-negative bacteremias. J. Chemother..

[8] Cunha B.A., McDermott B., Nausheen S. (2007). Single daily high-dose tigecycline therapy of a multidrug-resistant (MDR) *Klebsiella pneumoniae* and *Enterobacter aerogenes* nosocomial urinary tract infection. J. Chemother..

[9] Cunha B.A. (2017). Antibiotic Essentials.

[10] Woodford N., Hill R.L., Livermore D.M. (2007). In vitro activity of tigecycline against carbapenem-susceptible and -resistant isolates of *Klebsiella* spp. and *Enterobacter* spp.. J. Antimicrob. Chemother..

[11] Larson K.C., Belliveau P.P., Spooner L.M. (2011). Tigecycline for the treatment of severe Clostridium difficile infection. Ann. Pharm..

[12] Herpers B.L., Vlaminckx B., Burkhardt O., Blom H., Biemond-Moeniralam H.S., Hornef M., Welte T., Kuijper E.J. (2009). Intravenous tigecycline as adjunctive or alternative therapy for severe refractory Clostridium difficile infection. Clin. Inf. Dis..

[13] Britt N.S., Steed M.E., Potter E.M., Clough L.A. (2014). Tigecycline for the Treatment of Severe and Severe Complicated Clostridium difficile Infection. Inf. Dis. Ther..

[14] Baines S.D., Saxton K., Freeman J., Wilcox M.H. (2006). Tigecycline does not induce proliferation or cytotoxin production by epidemic Clostridium difficile strains in a human gut model. J. Antimicrob. Chemother..

[15] Prasad P., Sun J., Danner R.L., Natanson C. (2012). Excess deaths associated with tigecycline after approval based on noninferiority trials. Clin. Inf. Dis..

[16] Vasilev K., Reshedko G., Orasan R., Sanchez M., Teras J., Babinchak T., Dukart G., Cooper A., Dartois N., Gandjini H. (2008). A Phase 3, open-label, non-comparative study of tigecycline in the treatment of patients with selected serious infections due to-resistant Gram-negative organisms including *Enterobacter* species, *Acinetobacter baumannii* and *Klebsiella pneumoniae*. J. Antimicrob. Chem..

[17] Cheong E.Y., Gottlieb T. (2011). Intravenous tigecycline in the treatment of severe recurrent Clostridium difficile colitis. Med. J. Aust..

[18] Burkhardt O., Rauch K., Kaever V., Hadem J., Kielstein J.T., Welte T. (2009). Tigecycline possibly underdosed for the treatment of pneumonia: A pharmacokinetic viewpoint. Int. J. Antimicrob. Agents.

[19] Cunha B.A. (2015). Tigecycline dosing is critical in preventing tigecycline resistance because relative resistance is, in part, concentration dependent. Clin. Microbiol. Inf..

[20] De Pascale G., Montini L., Pennisi M., Bernini V., Maviglia R., Bello G., Spanu T., Tumbarello M., Antonelli M. (2014). High dose tigecycline in critically ill patients with severe infections due to multidrug-resistant bacteria. Crit. Care.

[21] Ramirez J., Dartois N., Gandjini H., Yan J.L., Korth-Bradley J., McGovern P.C. (2013). Randomized phase 2 trial to evaluate the clinical efficacy of two high-dosage tigecycline regimens versus imipenem-cilastatin for treatment of hospital-acquired pneumonia. Antimicrob. Agents Chemother..

[22] Falagas M.E., Vardakas K.Z., Tsiveriotis K.P., Triarides N.A., Tansarli G.S. (2014). Effectiveness and safety of high-dose tigecycline-containing regimens for the treatment of severe bacterial infections. Int. J. Antimicrob. Agents.

[23] Xie J., Wang T., Sun J., Chen S., Cai J., Zhang W., Dong H., Hu S., Zhang D., Wang X. (2014). Optimal tigecycline dosage regimen is urgently needed: Results from a pharmacokinetic/pharmacodynamic analysis of tigecycline by Monte Carlo simulation. Int. J. Inf. Dis..

[24] Garnacho-Montero J., Ferrandiz-Millon C. (2014). High dose of tigecycline for extremely-resistant Gram-negative pneumonia: Yes, we can. Crit. Care.

[25] DeRosa F.G., Corcione S., Di Perri G., Scaglione F. (2015). Re-defining tigecycline therapy. New Microbiol..

[26] Brust K., Evans A., Plemmons R. (2014). Favorable outcome in the treatment of carbapenem-resistant Enterobacteriaceae urinary tract infection with high-dose tigecycline. J. Antimicrob. Chemother..

